# Prognostic significance of natural killer cell depletion in predicting progressive fibrosing interstitial lung disease in idiopathic inflammatory myopathies

**DOI:** 10.3389/fimmu.2024.1404828

**Published:** 2024-04-30

**Authors:** Chenyi Shao, Nana Xia, Yan Zhen, Xueliang Zhang, Ninghui Yan, Qiang Guo

**Affiliations:** ^1^ Department of Rheumatology, Renji Hospital, Shanghai Jiao Tong University School of Medicine, Shanghai, China; ^2^ Jiading Branch, Ren Ji Hospital, Shanghai Jiao Tong University School of Medicine, Shanghai, China

**Keywords:** idiopathic inflammatory myopathy, progressive fibrosing interstitial lung disease, lymphocyte, natural killer cell, risk factor

## Abstract

**Objectives:**

Interstitial lung disease (ILD) is one of the common extramuscular involvement in idiopathic inflammatory myopathies (IIMs) (1). Several patients develop a progressive fibrosing ILD (PF-ILD) despite conventional treatment, resulting in a progressive deterioration in their quality of life (2). Here, we investigated the clinical and immune characteristics of IIM-ILD and risk factors for PF-ILD in IIM, mainly in anti-melanoma differentiation-associated protein 5 (anti-MDA5^+^) dermatomyositis (DM) and anti-synthetase syndrome (ASS).

**Methods:**

Here, a prospective cohort of 156 patients with IIM-ILD were included in the longitudinal analysis and divided into the PF-ILD (n=65) and non-PF-ILD (n=91) groups, and their baseline clinical characteristics were compared. Univariate and multivariate Cox analyses were performed to identify the variables significantly associated with pulmonary fibrosis progression in the total cohort, then anti-MDA5^+^ DM and ASS groups separately.

**Results:**

Peripheral blood lymphocyte counts, including T, B, and NK cell counts, were significantly lower in the PF-ILD group than in the non-PF-ILD group. This characteristic is also present in the comparison between patients with anti-MDA5^+^ DM and ASS. The multivariate Cox regression analysis revealed that age > 43.5 years [HR: 7.653 (95% CI: 2.005-29.204), p = 0.003], absolute NK cell count < 148 cells/μL [HR: 6.277 (95% CI: 1.572-25.067), p = 0.009] and absolute Th cell count < 533.2 cells/μL [HR: 4.703 (95% CI: 1.014-21.821), p = 0.048] were independent predictors of progressive fibrosing during 1-year follow-up for patients with anti-MDA5^+^ DM, while absolute count of NK cells < 303.3 cells/µL [HR: 19.962 (95% CI: 3.108-128.223), p = 0.002], absolute count of lymphocytes < 1.545×10^9^/L [HR: 9.684 (95% CI: 1.063-88.186), p = 0.044], and ferritin > 259.45 ng/mL [HR: 6 (95% CI: 1.116-32.256), p = 0.037] were independent predictors of PF-ILD for patients with ASS.

**Conclusions:**

Patients with anti-MDA5^+^ DM and ASS have independent risk factors for PF-ILD. Lymphocyte depletion (particularly NK cells) was significantly associated with PF-ILD within 1-year of follow-up for IIM-ILD

## Introduction

Idiopathic inflammatory myopathies (IIMs), a group of autoimmune systemic diseases with diverse clinical manifestations, can affect multiple parts of the body, including the skin, muscles, joints, and lungs (1); however, the lung is one of the most commonly involved extramuscular organs, with the reported prevalence ranging between 20% and 86% (2), associated with poor prognosis and increased mortality (3, 4). Most dermatomyositis (DM) (90%) and anti-synthetase syndrome (ASS) (70%–85%) cases with myositis-specific autoantibodies (MSAs) are usually associated with lung injury (5). Interstitial lung disease (ILD) is one of the most common pulmonary manifestations primarily characterized by inflammation and fibrosis of the lung tissue, which can occur before, after, or concurrently with cutaneous or muscular manifestations but typically occurs early in the disease course (6, 7). Despite conventional treatment targeting the underlying condition, a subset of patients with ILD continue to experience a progressive fibrotic phenotype characterized by inexorable deterioration of lung function, respiratory symptoms, signs on high-resolution computed tomography (HRCT), as well as higher early mortality rate, which can be described as progressive fibrosing interstitial lung disease (PF-ILD) (8–10). The introduction of the PF-ILD concept was associated with the clinical trial of an antifibrotic drug (11), and was progressively integrated into subsequent research, which suggested that individuals with ILDs other than IPF, including those with IIM-ILD and other connective tissue disease associated ILD (CTD-ILD) (12–14), are also at risk of developing PF-ILD. Although various clinical drug trials (11, 15) and experts (16) have proposed different criteria, the assessment predominantly relies on changes in lung function, radiological findings, and clinical symptomatology. The prevalence of PF-ILD in patients with CTDs is challenging to ascertain, which has been reported a range of 18%–44% in current studies with limited scope at present, including the groups of IIMs, rheumatoid arthritis, systemic sclerosis (SSc), and other CTDs (17–20). Further subdivision of IIMs according to few studies revealed that about 50% of patients with anti-MDA5^+^ DM developed PF-ILD (20), whereas 35% of those with ASS developed PF-ILD (12). As the prognosis of patients with PF-ILD is worse than that of those with stable and reversible ILD, early identification of IIM with comorbid progressive pulmonary fibrosis is crucial for clinical management and improving prognosis (7).

Despite generalizable risk factors, various cohort studies have also found predictive risk factors for progressive fibrosis specific to autoimmune ILDs. It has been proposed that patients with CTD with diabetes mellitus, steroid treatment, and a pattern of fibrosis on HRCT have a higher risk of developing PF-ILD in 2-year follow-up (21). Other laboratory and clinical indicators identified to predict rapid progression or poor prognosis include serum ferritin, C-reactive protein (CRP), lactate dehydrogenase (LDH), and Krebs Von den Lungen-6 (KL-6), as well as anti-melanoma differentiation-associated protein 5 (anti-MDA5) antibody titres, anti-Ro-52 levels, white blood cell (WBC) count, disease duration, fever, and age, although these studies mainly focused on patients with DM, particularly those with anti-MDA5^+^ (22–24).

Previous studies have found numerical or functional dysregulation of circulating natural killer (NK) cell plays a pivotal role in IIM-ILD (25, 26). One of our recent studies discovered that an amyopathic dermatomyositis-associated ILD group with a cluster of activated CD45RA^+^HLA-DR^+^CD8^+^ T cells and a reduced proportion of CD56^dim^ NK cells showed a high prevalence of rapidly progressive ILD and increased mortality rates (27), indicating that peripheral lymphocyte immunological profiles have predictable potential for the development of ILD fibrotic progression. Lin et al. also elucidated that in anti-MDA5^+^ DM patients, the significantly reduced levels and an inhibitory phenotype of peripheral NK cells are correlated with heightened disease activity and adverse prognosis (28).

Given the important role of NK cells in IIM-ILD, we aimed to explore the prognostic value of lymphocyte subsets, particularly NK cells, on ILD progression in a broader range of patients with IIM. Early identification of patients with PF-ILD is essential for clinical management. Therefore, we investigated the peripheral blood immunological characteristics and related variables in a group of patients with IIM, mainly containing two subgroups, anti-MDA5^+^ DM and ASS, to assess the risk of PF-ILD by exploring new immunological indices. This will help to identify early high-risk populations with fibrosing progression of ILD, guide more precise individualized therapy, and provide new insights for clinical treatment and prognostic assessment.

## Materials and methods

### Study design and patient selection

For this prospective cohort study, 185 patients with definite IIM-ILD were enrolled and followed up for 1 year between January 2020 and December 2022 at the Department of Rheumatology of the Renji Hospital of Shanghai Jiao Tong University. This study was conducted in accordance with the Declaration of Helsinki and approved by the Research Ethics Committee of Renji Hospital (ID:2013-126). All participants had provided informed consent for study enrolment and blood collection.

All participants fulfilled the classification criteria of the European League Against Rheumatism/American College of Rheumatology (29) and the 239th European Neuromuscular Centre International Workshop (30). The diagnosis of ILD is performed by radiologists and rheumatologists based on HRCT imaging (radiological signs of >10% lung affected, including ground glass, subpleural reticulation formation, traction bronchiectasis, and honeycombing) (31). Those with infection, cancer, other CTDs, and other chronic lung diseases were excluded from this study.

The diagnostic criteria for PF-ILD were based on the 2022 American Thoracic Society/European Respiratory Society/Japanese Respiratory Society/Latin American Thoracic Society (ATS/ERS/JRS/ALAT) guidelines (32). The group of PF-ILD was determined if at least two of the following three characteristics were met within 1 year (12 ± 2 months) of follow-up despite management: worsening of respiratory symptoms; functional deterioration, defined as a ≥5% absolute decrease in the percentage of predicted forced vital capacity (FVC% pred) or a ≥10% absolute decrease in predicted diffusion capacity for carbon monoxide (DLCO% pred); radiological deterioration, determined by an increase in traction bronchiectasis, reticular abnormalities, honeycombing, new ground-glass opacities, or increased lobar volume loss.

### Data collection

At baseline, all patients underwent symptom assessment, pulmonary function tests (PFTs), and HRCT scans. Simultaneously, peripheral blood samples were collected for lymphocyte subset tests and other serological marker evaluations. The results of these initial analyses did not influence decisions regarding individual treatment plans. Demographic and general information, including age at onset, sex, disease duration, clinical symptoms, and stable treatment regimens at baseline (glucocorticoids, intravenous immunoglobulin, antifibrotic agents, and immunosuppressants), were recorded. The results of the PFTs, including FVC% pred, forced expiratory volume in the first second (FEV1% pred), and DLCO% pred, were collected at baseline and follow-up endpoint. According to the radiologic characteristics of HRCT, histologic patterns, such as usual interstitial pneumonia (UIP), nonspecific interstitial pneumonia (NSIP), and organizing pneumonia (OP), were recognized (31). Concurrently, lesions observed radiologically were classified into inflammatory and fibrotic patterns (33).

Myositis-specific autoantibodies (MSAs), myositis-associated autoantibodies (MAAs), neutrophil-to-lymphocyte ratio (NLR), ferritin, LDH, creatine kinase (CK), KL-6, WBC count, CRP, erythrocyte sedimentation rate (ESR), alanine aminotransferase (ALT), and aspartate aminotransferase (AST) were evaluated by standardized methods in the clinical laboratory. MSAs and MAAs were tested using commercial line blots at baseline, including MDA5, aminoacyl-tRNA synthetase (ARS, including Jo-1, PL-7, PL-12, EJ, and OJ), Mi-2, TIFγ, NXP2, SAE, SRP, Ku, PM-Scl100/75, and Ro-52 (34). NLR was calculated using automated analyzers as follows: NLR = absolute neutrophil count/absolute lymphocyte count.

Lymphocyte subset tests was performed using flow cytometry and analyzed by the hospital’s clinical laboratory. Fresh blood samples were collected in anticoagulant tubes and delivered to our clinical laboratory within 2 hours at room temperature. The BD FACSCanto II flow cytometer (BD Biosciences, USA) was used to detect the proportions and absolute counts of lymphocyte subsets in peripheral blood, including total lymphocytes, CD19^+^ B cells, CD3^+^ T cells, CD3^+^CD4^+^ T cells, CD3^+^CD8^+^ T cells, and CD3^−^CD16^+^CD56^+^ NK cells.

### Statistical analysis

Statistical analysis and plotting were performed using R software (version 4.3.0), IBM SPSS Statistics for Windows, version 27.0 (IBM Corp., Armonk, N.Y., USA), and GraphPad Prism (version 9.3). Continuous variables are expressed as the mean with standard deviation or median with interquartile range according to data distribution, while categorical data are presented as frequencies with percentages. Comparisons between groups for continuous data were performed using the Student’s t-test or Mann–Whitney test. Categorical variables were compared using the chi-square or Fisher’s exact test, as appropriate. The PF-ILD optimal cut-off value was determined using the area under the curve (AUC) and Youden Index, while risk factors for PF-ILD were determined using univariate and multivariate Cox survival analyses. Biologically plausible clinical factors (p<0.05) in the univariate analysis were included in the multivariable analysis and adjusted for sex, age, and other confounders, presented as forest plots, and quantified using hazard ratio (HR) and 95% confidence interval (CI). Kaplan–Meier analysis was used to visualize the cumulative event-free survival rate. Statistical significance was set at p<0.05.

## Results

### Cohort characteristics

After excluding those 29 patients who lost HRCT or PFTs follow-up, 156/185 patients with IIM-ILD patients who had undergone both PFTs and HRCT tests at baseline and 1-year follow-up endpoint were included in the final longitudinal cohort (**Supplementary Figure S1**). The cohort showed a mean age of 53.19 years. Among them, 45 (28.8%) were male. During the follow-up, 65 (41.7%) patients met the deterioration criteria for PF-ILD, while 91 (59.3%) who had no ILD progression were classified as the non-PF-ILD group. Regarding disease subtypes, 60 (38.5%) patients had ASS, 84 (53.8%) had anti-MDA5^+^DM, and 12 (7.7%) had other positive antibodies, including Mi-2, TIFγ, NXP2, SAE, PM-Scl75. The predominant HRCT pattern was NSIP, observed in 131 (84.0%) patients, while 22 (14.1%) had UIP, and only 3 (1.9%) had OP. All participants were treated with glucocorticoids (GC) at baseline, with a median maximum dose of 40 mg/day. In addition to GC, a significant proportion of patients (92.3%) received immunosuppressants or biologics. Intravenous immunoglobulin (IVIG) was administered to 8.3% of the participants. Furthermore, 17.9% of the patients received antifibrotic therapy.

### Baseline characteristics of IIM patients by median NK cell count

The median baseline absolute NK cell count was 173.5 cells/µL (IQR 87.97–318.85). Patients with absolute NK cell counts below the median level at baseline had a relatively shorter disease course and a higher incidence of PF-ILD. Additionally, there were more anti-MDA5^+^DM patients in this group. These patients also exhibited a decrease in other lymphocyte subsets, including B cells, T cells, Th cells, Ts cells, and WBC; had elevated levels of ALT, AST, CRP, ESR, NLR, and ferritin compared to patients with NK cell counts above the median. Patients with low NK cell counts more frequently presented with fever, heliotrope rash, and Gottron’s sign. They also required higher doses of glucocorticoids and more frequent use of immunosuppressants, particularly tofacitinib (TOF). In contrast, patients with high NK cell counts were more likely to be treated with methotrexate (MTX) and rituximab (RTX) (all p<0.05) (**Supplementary Table S1**).

### Baseline characteristics in total cohort: PF-ILD vs non-PF-ILD

**Figure 1:**
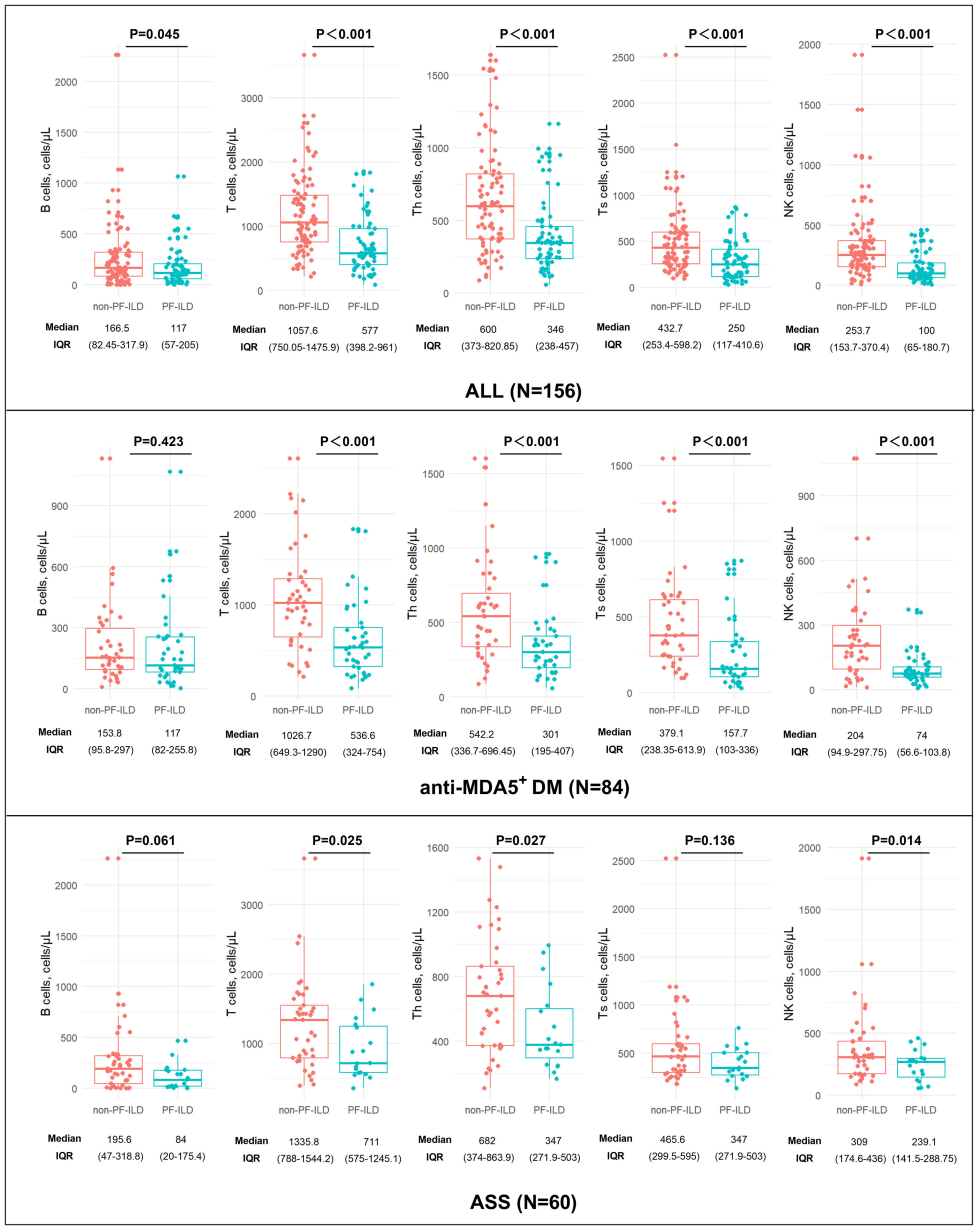
Comparison of lymphocyte counts between PF-ILD and non-PF-ILD groups.

In the total cohort, the absolute number of peripheral blood lymphocytes at baseline, including B, T, Th, Ts, and NK cells decreased significantly in the PF-ILD group. The median absolute number of NK cells was also significantly lower in the PF-ILD group in both anti-MDA5^+^DM [74 (IQR 56.6-103.8) vs 204 (94.9-297.75); P < 0.001] and ASS [239.1 (IQR 141.5-288.75) vs 309 (174.6-436); P < 0.001], as were the levels of T cells and Th cells. The level of B cells and Ts cells was decreased in anti-MDA5^+^DM PF-ILD group, but not in ASS PF-ILD group (**Figure 1**).

The results of comparisons of baseline parameters between the PF-ILD and non-PF-ILD groups in total follow-up cohort are summarized in **Supplementary Table S2**. Patients with PF-ILD had no statistically significant differences in FVC, FEV1, and DLCO at baseline (p>0.05) but had significantly lower FVC, FEV1, and DLCO at the endpoint than those without PF-ILD (p<0.001). The absolute number of peripheral blood lymphocytes at baseline, including B, T, Th, Ts, and NK cells and WBCs, decreased significantly in the PF-ILD group; however, the NLR was significantly elevated. Additionally, the PF-ILD group showed significantly higher ferritin, ALT, and AST levels than the control group (all p<0.05). However, no statistical difference was found in baseline clinical symptoms or baseline treatment (p>0.05).

**Supplementary Table S3** presents the results of univariate and multivariable Cox regression analyses for various factors potentially associated with PF-ILD in the total cohort. In the univariate Cox regression analysis, age, disease course, and several peripheral lymphocyte subset parameters, including T cells, Th cells, Ts cells, and NK cells, were significantly associated with the progression of pulmonary fibrosis in IIM-ILD patients. However, in the multivariable Cox regression analysis, after adjusting for age, sex, and other covariates, only the absolute NK cell count remained a strong independent predictor of pulmonary fibrosis progression within one year [HR: 0.995 (95% CI: 0.992–0.997), p < 0.001].

### The differences between patients with anti-MDA5^+^ DM and ASS

In our follow-up cohort, most patients had anti-MDA5^+^ DM (n=84, 53.8%) and ASS (n=60, 38.5%), with some differences in disease characteristics between the two subtypes. Comparing the characteristics of these two IIM subgroups (**Table 1**), patients with anti-MDA5^+^ DM had PF-ILD more frequently than those with ASS (48.8% vs 31.7%, p=0.04). The analysis of HRCT patterns revealed that NSIP predominated in both groups, being present in 75% of the ASS cohort and 89.3% of the anti-MDA5^+^DM group, as well as a higher proportion of UIP observed in ASS. When analyzing the inflammatory or fibrotic patterns, the proportion of fibrotic patterns was significantly lower in anti-MDA5^+^DM, suggesting a predominance of inflammatory patterns in this group. Patients with anti-MDA5^+^ DM had a lower average onset age and shorter times from onset to diagnosis than those with ASS. A significantly lower number of peripheral blood lymphocytes, including B, T, Th, Ts, and NK cells and total lymphocytes, were observed in anti-MDA5^+^ DM, as well as WBC and neutrophils. Moreover, we found that anti-MDA5^+^ DM had higher ferritin, ALT, AST, and ESR levels, as well as an increased incidence of heliotrope rash and Gottron sign, but lower CK and CRP. All patients in the cohort were treated with glucocorticoids, although those with anti-MDA5^+^ had been prescribed higher dosages of glucocorticoids. Patients with anti-MDA5^+^ DM were treated with a higher frequency of tacrolimus and tofacitinib, while those with ASS were treated with methotrexate, cyclophosphamide, and rituximab (all p<0.05).

**Table 1:** The differences between anti-MDA5^+^ DM and ASS patients.

	ASS (n=60)	anti-MDA5^+^ DM (n=84)	P value
age, mean (SD), years	55.32 (11.34)	50.77 (10.36)	0.015*
Male, n (%)	17 (28.3)	25 (29.8)	0.852
Course, month	6.5 (3-16.25)	4 (1.75-8.25)	0.042*
Follow-up duration, month	10 (10-11.25)	10 (6-11)	0.091*
PF-ILD, n (%)	19 (31.7)	41 (48.8)	0.040*
**HRCT pattern, %**			0.042*
NSIP	45 (75.0)	75 (89.3)	
UIP	14 (23.3)	7 (8.3)	
OP	1 (1.7)	2 (2.4)	
**Fibrotic pattern, n (%)**	29 (48.3)	24 (28.6)	0.038*
PFT			
FVC % pred at baseline, mean (SD)	64.81 (15.5)	71.47 (19.26)	0.023*
FEV1% pred at baseline, mean (SD)	68.31 (15.44)	72.84 (18.09)	0.109
DLCO% pred at baseline mean (SD)	40.29 (16.11)	48.28 (19.07)	0.007**
Peripheral lymphocyte subset test			
B cells, %	9 (1.75-19.4)	15.62 (8.51-25.59)	< 0.001***
T cells, %	67.45 (59.43-74.51)	68.76 (61-76.28)	0.711
Th cells, mean (SD), %	35.26 (9.37)	38.76 (12.19)	0.053
Ts cells, %	28.05 (23-35.19)	24.29 (18-34.19)	0.103
CD4/CD8	1.22 (0.89-1.57)	1.56 (0.95-2.44)	0.032*
NK cells, %	17.15 (11-25.31)	10.7 (6.56-19.31)	< 0.001***
B cells, cells/µL	144.25 (24.5-282.32)	144.5 (86.8-259.72)	0.372
T cells, cells/µL	1049.4 (704-1488.02)	739.5 (419.05-1123.25)	< 0.001***
Th cells, cells/µL	568.85 (356.45-842.38)	376.45 (243-608.32)	0.002**
Ts cells, cells/µL	449.2 (291.45-581.6)	279.15 (132.4-506.95)	< 0.001***
NK cells, cells/µL	288.75 (168.76-369.58)	102.4 (62.93-209.25)	< 0.001***
Lymphocytes, ×10^9^/L	1.57 (1.04-2.01)	0.95 (0.64-1.54)	< 0.001***
WBC, ×10^9^/L	11.02 (8.59-13.24)	6.62 (5.02-8.37)	< 0.001***
Neutrophils, ×10^9^/L	8.07 (6.19-11.05)	5.2 (3.47-6.28)	< 0.001***
NLR	4.75 (3.4-9.08)	4.49 (3.21-8.47)	0.398
laboratory tests			
Ferritin, ng/mL	145 (71.53-406.4)	443.6 (77.35-905.42)	0.004**
LDH, IU/L	252 (214.5-310.25)	271 (215-334.25)	0.235
CK, IU/L	50.5 (38.25-135.25)	42.5 (30-60.25)	0.010*
KL-6, IU/mL	1395 (749-2289.25)	1009 (631.5-2190.75)	0.451
ALT, IU/L	30 (16-44)	37 (19-78.25)	0.046*
AST, IU/L	25.5 (20-35.25)	33.5 (22-57.75)	0.003**
CRP, mg/L	3.07 (1.04-9.55)	1.89 (0.5-3.15)	0.001**
ESR, mm/h	12 (5-22.25)	18 (11-29.5)	0.003**
clinical manifestation, n (%)			
Fever	5 (8.3)	17 (20.2)	0.0502
Cough	25 (41.7)	26 (31)	0.185
Heliotrope rash	6 (10)	41 (48.8)	< 0.001***
Gottron sign	12 (20)	60 (71.4)	< 0.001***
Mechanism’s hands	17 (28.3)	18 (21.4)	0.341
Arthritis	8 (13.3)	22 (26.2)	0.061
Treatment			
antifibrotics	10 (16.7)	17 (20.2)	0.588
maximum GC dose, mg/day	40 (15-60)	50 (25-80)	0.025*
IVIG, n (%)	4 (6.7)	8 (9.5)	0.541
Immunosuppressants or biologics, n (%)	53 (88.3)	79 (94)	0.221
TAC	5 (8.3)	18 (21.4)	0.034*
CsA	3 (5)	0 (0)	0.139
MMF	3 (5)	0 (0)	0.139
CTX	8 (13.3)	1 (1.2)	0.009**
Agu	2 (3.3)	0 (0)	0.172
MTX	10 (16.7)	2 (2.4)	0.002**
TOF	14 (23.3)	72 (85.7)	< 0.001***
Baricitinib	0 (0)	1 (1.2)	1
RTX	19 (31.7)	2 (2.4)	< 0.001***
Tocilizumab	2 (3.3)	1 (1.2)	0.767

Except where otherwise indicated, values are shown as the medians (interquartile range). *p < 0.05; **p < 0.01; ***p < 0.001.

NSIP, nonspecific interstitial pneumonia, UIP, usual interstitial pneumonia, OP, organizing pneumonia; CK, creatine kinase; KL-6, Krebs von den Lungen-6; ALT, alanine transaminase; AST, aspartate transaminase; anti-ARS: anti-aminoacyl-tRNA synthetase; CRP, C-reactive protein; DM: dermatomyositis; ESR, erythrocyte sedimentation rate; GC, glucocorticoid; antifibrotics, including nintedanib and pirfenidone; IVIG, intravenous immunoglobulin; immunosuppressants or biologics, including tacrolimus (TAC), cyclosporin A (CSA), and mycophenolate mofetil (MMF); Cyclophosphamide (CTX), azathioprine (AZA), methotrexate (MTX), tofacitinib (TOF), Baricitinib, Rituximab (RTX) and tocilizumab; LDH, lactate dehydrogenase; NLR, neutrophil-to-lymphocyte ratio; MSAs: Myositis-specific autoantibodies; anti-MDA5: anti–melanoma differentiation–associated protein 5; PFT: pulmonary function test; FVC% pred: percentage of predicted forced vital capacity; FEV1% pred: percentage of forced expiratory volume in the first second; DLCO% pred: percentage of the predicted diffusion capacity for carbon monoxide.

### Risk factors of PF-ILD in anti-MDA5^+^ DM and ASS

Receiver operating characteristic (ROC) curve analysis and univariate and multivariate regression analyses were performed to determine the risk factors for patients with PF-ILD in both two subgroups.

**Figure 2:**
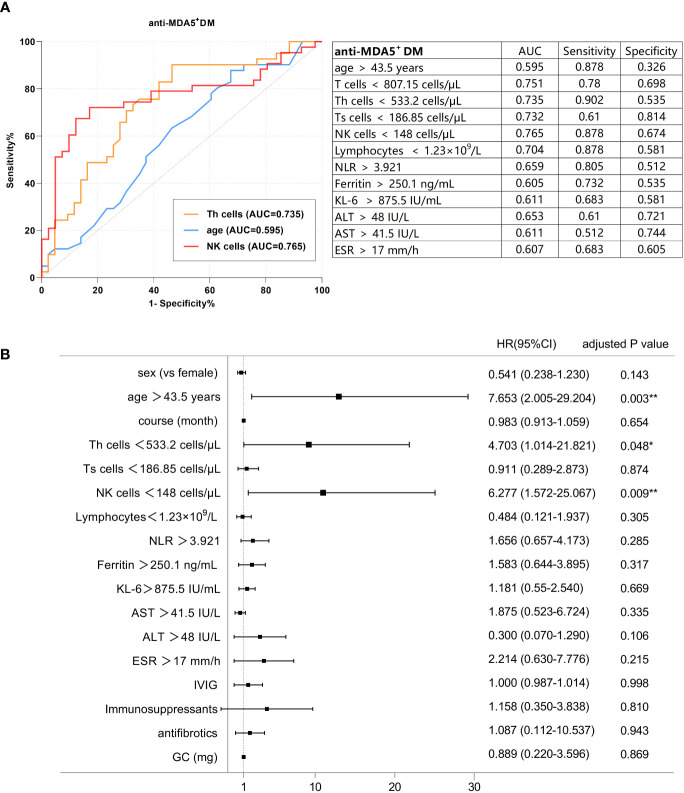
**(A)** ROC analysis of the baseline factors of PF-ILD in anti-MDA5^+^ DM. **(B)** Forest plot of multivariate Cox analysis in anti-MDA5^+^ DM.

In anti-MDA5^+^ DM, the best cut-off values for various parameters are shown in **Figure 2A**. Subsequently, candidate parameters identified in univariate Cox regression analyses (**Supplementary Table S4**) were included in multivariate analysis and later adjusted for covariates (**Figure 2B**). As revealed in univariate analyses, in the anti-MDA5^+^ DM group, age > 43.5 years, shorter disease course, a decreased number of peripheral T cells, Th cells, Ts cells, NK cells, and lymphocytes, as well as increased levels of KL-6, AST, ALT, NLR, and ESR were significantly associated with PF-ILD. However, in further multivariate regression analysis with the adjustment of other covariates, only age > 43.5 years [HR: 7.653 (95% CI: 2.005-29.204), p = 0.003], absolute NK cell count < 148 cells/μL [HR: 6.277 (95% CI: 1.572-25.067), p = 0.009] and Th cells < 533.2 cells/μL [HR: 4.703 (95% CI: 1.014-21.821), p = 0.048] remained significant independent predictors of PF-ILD in patients with anti-MDA5^+^ DM. Other factors, such as clinical manifestations and treatment modalities, did not show significant associations with PF-ILD in the multivariable analysis.

The optimal cut-off values for the potential poor prognostic factors at baseline associated with fibrosing progression included the absolute count of NK cells < 148 cells/μL [AUC, 0.765; sensitivity, 0.878; and specificity, 0.674], absolute count of Th cells < 533.2 cells/μL [AUC, 0.735; sensitivity, 0.902; and specificity, 0.535], and age > 43.5 years [AUC, 0.595; sensitivity, 0.878; and specificity, 0.326]. The cut-off values for individual biomarkers were rounded for further analyses.

**Figure 3:**
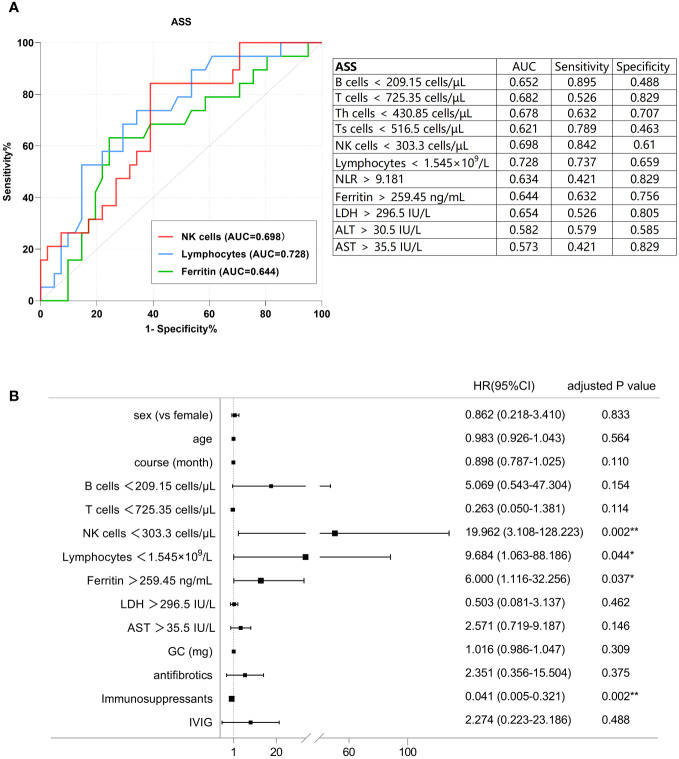
**(A)** ROC analysis of the baseline factors of PF-ILD in ASS. **(B)** Forest plot of multivariate Cox analysis in ASS.

**Figure 3A** presents the ROC analysis of risk factors in patients with ASS, and the correlation between the variables of interest and the incidence of PF-ILD was assessed using univariate Cox regression analysis (**Supplementary Table S5**). In patients with ASS, decreased number of B cells, T cells, NK cells, and lymphocytes, as well as increased levels of ferritin, LDH, NLR, and AST were risk factors for PF-ILD in the univariate analysis. Subsequently, the results of ROC and multivariate regression analyses demonstrated the following independent risk factors: absolute count of NK cells < 303.3 cells/µL [HR: 19.962 (95% CI: 3.108-128.223), p = 0.002], absolute count of lymphocytes 1.545×10^9^/L [HR: 9.684 (95% CI: 1.063-88.186), p = 0.044], and ferritin > 259.45 ng/mL [HR: 6 (95% CI: 1.116-32.256), p = 0.037] (**Figure 3B**).

The optimal cut-off values for the potential poor prognostic factors at baseline associated with fibrosing progression were absolute count of NK cells < 303.3 cells/µL [AUC, 0.698; sensitivity, 0.842; and specificity, 0.61], absolute lymphocyte count < 1.545×10^9^/L [AUC, 0.728; sensitivity, 0.737; and specificity, 0.659], ferritin >259.45 IU/L [AUC, 0.644; sensitivity, 0.632; and specificity, 0.756].

**Table 2** presents the results of univariate and multivariate Cox regression analyses using NK cell count as both a continuous and binary variable. After adjusting for various covariates using three different models, NK cell count consistently remained a significant risk factor for predicting PF-ILD in both disease subtypes.

**Table 2:** Cox regression analyses of NK cell count.

	anti-MDA5^+^ DM (n=84)		ASS (n=60)	
	HR (95% CI)	P value	HR (95% CI)	P value
Univariate Cox regression				
Continuous NK cell count	0.993 (0.989-0.997)	<0.001***	0.996 (0.992-1)	0.035*
Binary NK cell count	6.838 (2.668-17.525)	<0.001***	5.816 (1.687-20.053)	0.005**
Multivariable Cox regression(Binary NK cell count)				
Model A	5.928 (1.994-17.625)	0.001**	8.324 (2.112-32.807)	0.002**
Model B	6.811 (1.875-24.747)	0.004**	7.683 (1.854-31.85)	0.005**
Model C	6.277 (1.572-25.067)	0.009**	19.962 (3.108-128.223)	0.002**

*p < 0.05; **p < 0.01; ***p < 0.001. In the anti-MDA5^+^DM group, binary NK cell count was based on the optimal cut-off (148 cells/µL); Then Model A was adjusted for age, sex, and disease course; Model B further included ferritin, KL-6, ALT, AST, and ESR in addition to the variables in Model A; and Model C incorporated immunotherapy on top of the variables in Model B. For the ASS group, binary NK cell count was based on the optimal cut-off (303.3 cells/µL); Then Model A was adjusted for age, sex, and disease course; Model B added ferritin, LDH, and AST to the variables in Model A; and Model C included immunotherapy in addition to the variables in Model B.

**Figure 4:**
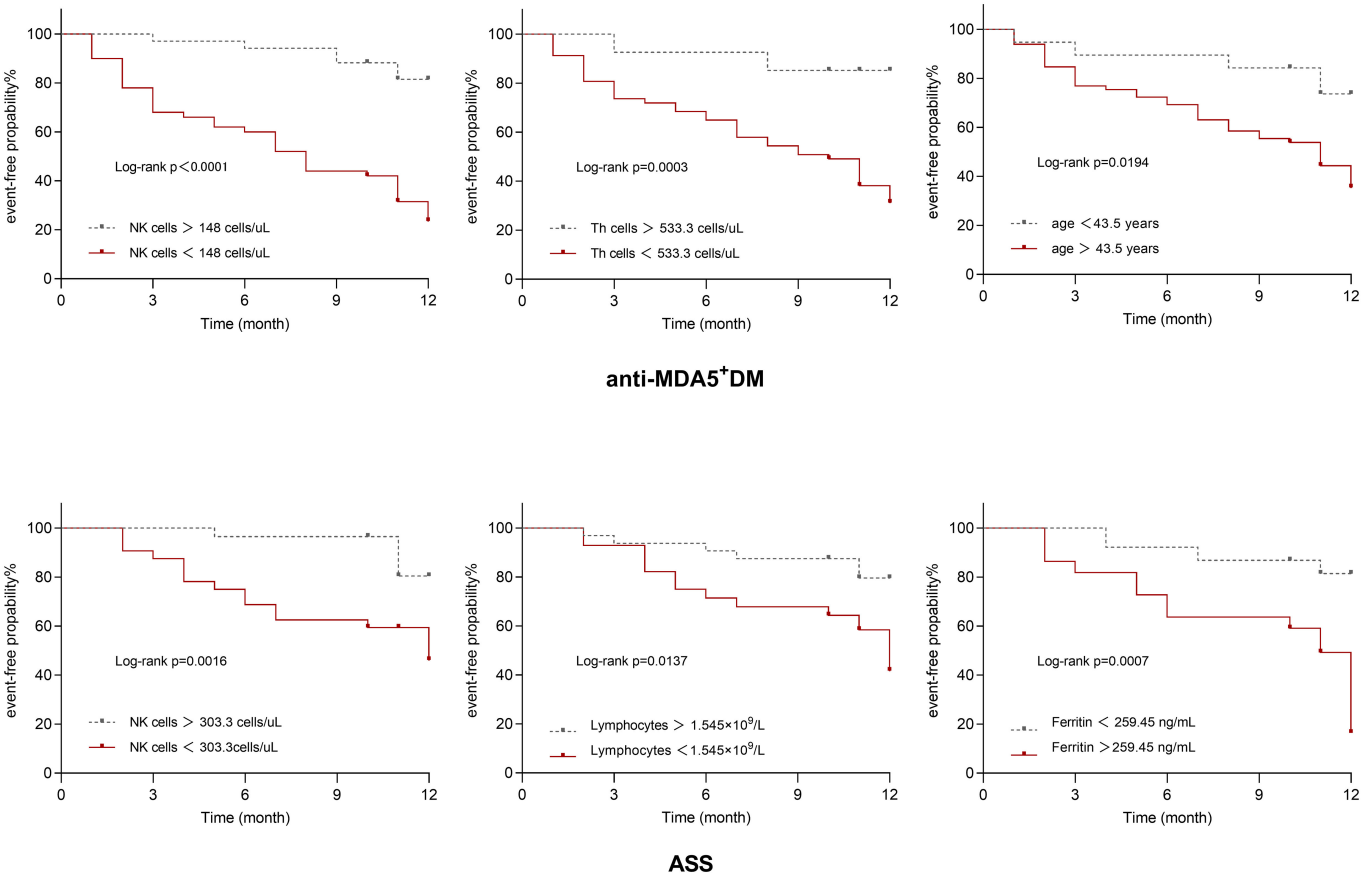
Kaplan–Meier survival curves for anti-MDA5^+^DM and ASS.

The Kaplan-Meier survival plots presented in **Figure 4** depict event-free probability over time after the two patient groups were grouped according to their ultimately statistically significant risk factors. Patients were divided according to optimal cut-off values of different variables. Anti-MDA5^+^DM patients with lower counts of NK cells, lower counts of Th cells, and older age had a higher probability of experiencing PF-ILD, while ASS patients with depletion in NK cells and Th cells, and elevated levels of ferritin also demonstrate a higher probability of developing PF-ILD (log-rank test, P < 0.05).

## Discussion

Although evidence suggests the potential value of lymphocytes in the progression and prognosis of autoimmune disease-associated ILD, related studies are limited (27, 35). In this prospective study, we systematically described the clinical and immune characteristics of patients with IIM-ILD, with a focus on lung involvement, and separately discussed the two subpopulations, anti-MDA5^+^ DM and ASS, which are most susceptible to ILD; the relationships among peripheral blood lymphocyte characteristics, clinical parameters, and disease progression were also discussed. We found that immune cell depletion was strongly associated with pulmonary fibrosis progression in patients with IIM-ILD, and the lymphocyte characteristics of different IIM subtypes were partly different. The depletion of NK and Th cells and older age of onset were associated with PF-ILD in patients with anti-MDA5^+^ DM, whereas lymphopenia, NK cell depletion, and high levels of ferritin in the peripheral blood of patients with ASS were associated with PF-ILD. To the best of our knowledge, this is the first prospective study to focus on the immunological subtypes as risk factors of PF-ILD in a larger group of patients with IIM, particularly the two different IIM subtypes, and we identified the potential value of lymphocytes (especially NK cells) in predicting PF-ILD.

The ‘PF-ILD’ concept is more appropriate for the broader IIM population than the short-term assessed ‘rapidly progressive ILD (RP-ILD)’, favoring the exploration of more meaningful indicators by reducing bias from critically ill patients. In our study, PF-ILD occurred in approximately 40% of patients with IIM, which is slightly higher than other related studies. First, similar CTD-ILD studies associated with PF-ILD were notably limited, most of which did not explicitly analyze each disease subtype; second, most patients in our cohort were those with anti-ARS^+^ and anti-MDA5^+^, which can be supported by studies that found approximately a 50% probability of PF-ILD in anti-MDA5^+^ DM (20).

Currently, it is well known that risk factors for developing ILD during IIM are mainly associated with MSAs (such as MDA5) and ARS (36), and each IIM subgroup has its pathophysiology. Thus, we separately analyzed the immune profiles of anti-MDA5^+^ DM and ASS. Based on our findings, individuals with anti-MDA5^+^ DM had a significantly higher probability of developing PF-ILD than those with ASS; additionally, they have a higher likelihood of having classic rashes, such as heliotrope rash and Gottron sign and a tendency to develop fever and arthritis. Moreover, individuals with anti-MDA5^+^ DM have more severe lymphocyte depletion than those with ASS, as well as higher levels of liver enzymes and ferritin, which are similar to the clinical characteristics of viral infection (37). These findings reflect differences in the pathogenesis of the two IIM-ILD groups with different antibodies; however, the risk factors for PF-ILD in anti-MDA5^+^ DM and ASS were somewhat similar.

Our results suggest that NK cell depletion was the significant risk factor for PF-ILD within 1 year in both patients with anti-MDA5^+^ DM and ASS, providing strong evidence for the association between NK cells and IIM-ILD and revealing the potential role of NK cell count as a predictor of pulmonary fibrotic progression. Nevertheless, the optimal cutoffs are different, with a lower cutoff in the anti-MDA5^+^ DM group, whereas the cutoff for ASS is within the normal range of NK cells, possibly because of differences in sample sizes that require further expansion. NK cells are natural effector cells involved in innate immunity and are currently considered to be involved in regulating adaptive immunity, playing an important role in various immunological diseases (38). However, the potential of NK cells in IIM appears to be underestimated, and related research is limited. Several studies have found that NK cell depletion is more noticeable in patients with ASS with severe lung involvement (25) and in those with anti-MDA5^+^ DM than in those with other IIM subtypes (28). Our results not only supported that patients with anti-MDA5^+^ DM show more pronounced depletion of NK cells but also indicated that the number of NK cells can be used to identify patient populations more prone to progressive exacerbations of ILD in anti-MDA5^+^ DM and ASS. Additionally, comparing the characteristics of the lower NK cell counts group with the higher NK cell counts group within the overall IIM cohort, it was observed that patients with NK cell counts below the median not only exhibited a significant increase in the incidence of PF-ILD but also showed a heightened risk of RP-ILD. This aligned with the findings of Ye’s study (27), which reported a higher proportion of RP-ILD in patients with a reduction in CD56^dim^ NK cells accompanied by a cluster of activated CD45RA^+^HLA^-^DR^+^CD8^+^ T cells. Our findings, therefore, reinforced the significant role of NK cells in evaluating and predicting the progression of ILD.

It is suggested that NK cells in the peripheral circulation play an inhibitory role in disease progression by coordinating the extent of the inflammatory response (39), whereas a reduction in circulating NK cells may weaken the suppression of pathogenic immune cells. Nevertheless, the exact mechanism of the NK cell depletion in IIM remains unknown. One hypothesis is that the depletion of circulating NK cells may be due to their migration from the peripheral circulation to the local site or apoptosis, reported by related clinical studies about ASS (26), SSc (40, 41), and sarcoidosis (42). Notably, the migration of NK cells to the lungs in anti-MDA5^+^ DM patients may be driven by the local production of chemokines induced by IFN-γ, such as CXCL10 (43, 44). Once recruited to the lungs, NK cells can further secrete IFN-γ, creating a positive feedback loop that amplifies the inflammatory response and exacerbates tissue damage (45). NK cell dysregulation has been reported in studies related to the COVID-19 infection, which inferred that direct viral attack, hyperinflammatory responses, activation-induced cell death, and the mobilization/homing of NK cells to affected tissues may be responsible for the NK cell depletion (46). However, these speculative mechanisms require further studies to elucidate the precise role of NK cells and IFN-γ in the pathogenesis of IIM-ILD.

Previous studies have found lymphocyte dysregulation as a risk factor for acute progression or death in anti-MDA5^+^ DM. For example, Zuo et al. demonstrated that reduced levels of CD3^+^, CD3^+^CD4^+^, and CD3^+^CD8^+^ T cells are predictors for the development of rapid progressive ILD in anti-MDA5^+^ DM (35), which was partially verified in our study. We also found a significant decrease in the levels of T, Th, Ts, and B cells and total lymphocytes in patients with PF-ILD; additionally, a decreased number of Th cells was found correlated with the risk of PF-ILD in patients with anti-MDA5^+^ DM. In contrast, lymphocyte depletion was also the significant risk factor for PF-ILD in patients with ASS, highlighting the important function and study prospect of lymphocytes in IIM-ILD.

Many studies have confirmed that laboratory markers, such as anti-MDA5 antibody, KL-6, ferritin, LDH, CK, CRP, and NLR, may serve as diagnostic and/or prognostic biomarkers of disease activity in IIM-ILD (47–53). Hyperferritinaemia has been recognized as an independent risk factor for poor prognosis in patients with anti-MDA5^+^ IIM-ILD (35, 49, 54) and in those with ASS-ILD (55). Ferritin is associated with macrophage activation (56). Our study revealed the predictive value of hyperferritinaemia for PF-ILD in patients with ASS. Given that our assessment criteria differ from death or acute exacerbation within 3 months, the results of multivariate regression analysis of anti-MDA5^+^ DM showed no statistical significance of high ferritin as an independent risk factor.

Furthermore, our study showed that patients with anti-MDA5^+^ or anti-ARS^+^ commonly exhibit comorbidity with anti-Ro52 antibodies because Ro52 forms Ro52/IgG/HLA-DR complexes on the cell surface, which are specifically recognized by autoantibodies in some patients with inflammatory myopathies (57).

While the vast majority of patients were treated with either glucocorticoids or immunosuppressive therapy at enrollment, our analysis revealed no substantial differences in the specifics of immunological therapy or antifibrotic treatment between the PF-ILD and non-PF-ILD groups at baseline. This finding suggests that the initial use of immunological therapy may not serve as a direct risk factor for PF-ILD. However, it is important to note that in the multivariate Cox regression analysis, immunosuppressant use appeared to emerge as an independent protective factor for prognosis in PF-ILD patients in ASS, despite showing no significance in the univariate analysis. However, due to the wide variety of immunosuppressants and the limited number of cases using each immunosuppressant, it is challenging to further analyze the impact of different immunosuppressants on prognosis.

This study had some limitations. Firstly, as a single-center study with a limited sample size, further multicenter studies with larger sample sizes and external validation are required to confirm these findings and explore the characteristics of more disease subtypes. Secondly, the heterogeneity of NK cells in different autoimmune diseases (58) and the potential impact of glucocorticoids and immunosuppressive treatments may have masked differences in clinical or laboratory test results. Furthermore, the functions and mechanisms of various lymphocyte subtypes in IIM-ILD were not thoroughly investigated.

Studies with larger sample sizes and more refined experimental designs are needed to dynamically record patients’ use of immunosuppressants during disease progression to perform a more accurate evaluation of the impact of different therapies on the prognosis of PF-ILD patients. Additionally, investigating the potential impact of immunosuppressants on lymphocyte subsets is crucial for comprehensively understanding the mechanisms of PF-ILD. Future research should also focus more on the functions of these immune subtypes to elucidate their significance in disease progression, particularly in different disease subtypes.

## Conclusion

Patients with anti-MDA5^+^ DM and ASS have different immune profiles, and they have their independent risk factors for PF-ILD; however, both groups have components associated with lymphocyte exhaustion. The depletion of NK cells with different cutoff values is a risk factor for PF-ILD in these two disease subtypes, suggesting that NK cell count may provide valuable information for the prognostic assessment of patients with IIM-ILD, thereby improving our understanding of disease pathogenesis. Our findings indicate that patients at risk of PF-ILD can be identified earlier by testing the NK cell and other lymphocyte levels, which could help clinicians manage patients with IIM-ILD and have implications for clinical monitoring, individualized therapy, and pathogenesis studies. Future studies are needed to focus on the functions and mechanisms of NK cell depletion and other cell subsets in IIM-ILD.

## Data availability statement

The original contributions presented in the study are included in the article/**Supplementary Material**, further inquiries can be directed to the corresponding author.

## Ethics statement

The studies involving humans were approved by Research Ethics Committee of Renji Hospital. The studies were conducted in accordance with the local legislation and institutional requirements. The participants provided their written informed consent to participate in this study.

## Author contributions

CS: Conceptualization, Formal analysis, Investigation, Methodology, Writing – original draft, Writing – review & editing, Data curation, Software, Visualization. NX: Conceptualization, Data curation, Formal analysis, Investigation, Methodology, Software, Visualization, Writing – original draft, Writing – review & editing. YZ: Data curation, Investigation, Methodology, Software, Visualization, Writing – original draft, Writing – review & editing. XZ: Data curation, Investigation, Visualization, Writing – original draft, Writing – review & editing, Supervision. NY: Data curation, Investigation, Supervision, Writing – original draft, Writing – review & editing. QG: Investigation, Supervision, Writing – original draft, Writing – review & editing, Conceptualization, Formal analysis, Funding acquisition, Methodology, Project administration, Resources.
