# Supplementary material for: Prognostic significance of natural killer cell depletion in predicting progressive fibrosing interstitial lung disease in idiopathic inflammatory myopathies
Source: Front Immunol. 2024 Apr 30;15:1404828. doi: 10.3389/fimmu.2024.1404828 (PMC11091831; doi:10.3389/fimmu.2024.1404828)
Supplement: Supplementary file 1 [file DataSheet_1.docx]

Supplementary Figure S 1 Flow chart of the current study


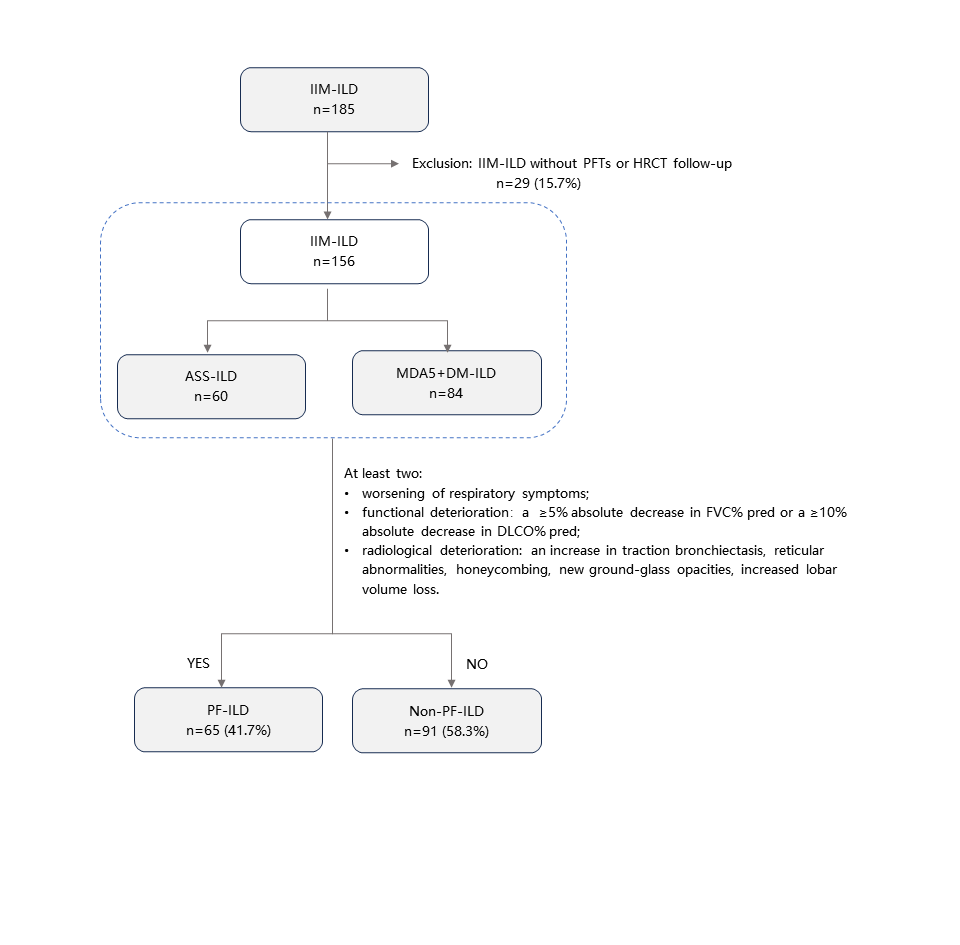


Supplementary Table S1 Baseline clinical and biochemical characteristics of IIM patients by median NK cell count.

| characteristics | **IIM ILD（n=156）** | **NK cells＞173.5 cells/μL（n=78）** | **NK cells＜173.5 cells/μL（n=78）** | **p value** |
| --- | --- | --- | --- | --- |
| age, mean (SD), years | 53.19 (11.09) | 54.56 (10.42) | 51.82 (11.62) | 0.123 |
| Male, n (%) | 45 (28.8) | 23 (29.5) | 22 (28.2) | 0.86 |
| Course, month | 6 (2-15) | 7 (4-16) | 4 (1-7.75) | ＜0.001*** |
| Follow-up duration, month | 5 (2-13) | 10 (10-12) | 10 (4-10) | ＜0.001*** |
| PF-ILD, n (%) | 65 (41.7) | 18 (23.1) | 47 (60.3) | ＜0.001 |
| RP_ILD, n (%) | 21 (13.5) | 3 (3.8) | 18 (23.1) | ＜0.001*** |
| **disease subtype, n (%)** |  |  |  | ＜0.001 |
| ASS | 60 (38.5) | 43 (55.1) | 17 (21.8) |  |
| Anti-MDA5^+^DM | 84 (53.8) | 28 (35.9) | 56 (71.8) |  |
| other subtype | 12 (7.7) | 7 (9.0) | 5 (6.4) |  |
| **MSAs** |  |  |  |  |
| Anti-MDA5 | 84 (53.8) | 28 (35.9) | 56 (71.8) | ＜0.001 |
| Anti-ARS | 60 (38.5) | 43 (55.1) | 17 (21.8) | ＜0.001 |
| Anti-Ro52 | 52 (33.3) | 26 (33.3) | 26 (33.3) | 1 |
| **HRCT pattern, n (%)** |  |  |  | 0.064 |
| NSIP | 131 (84.0) | 61 (78.2) | 70 (89.7) |  |
| UIP | 22 (14.1) | 16 (20.5) | 6 (7.7) |  |
| OP | 3 (1.9) | 1 (1.5) | 2 (2.2) |  |
| **Fibrotic pattern, n (%)** | 56 (35.9) | 31 (39.7) | 25 (32.1) | 0.317 |
| **PFT** |  |  |  |  |
| FVC % pred at baseline, mean (SD) | 69.34 (18.75) | 69.53 (16.89) | 68.65 (18.59) | 0.755 |
| FEV1% pred at baseline, mean (SD) | 71.71 (18.01) | 72.22 (16.36) | 70 (17.36) | 0.414 |
| DLCO% pred at baseline mean (SD) | 46.34 (19.48) | 44.74 (16.12) | 45.41 (19.95) | 0.817 |
| FVC% pred at endpoint, mean (SD) | 69.27 (19.71） | 72.42 (18.91) | 66.13 (20.12) | 0.046* |
| FEV1% pred at endpoint, mean (SD) | 71.99 (20.20) | 74.76 (19.45) | 69.21 (20.68) | 0.086 |
| DLCO% pred at endpoint, % | 48.45 (36.55-60.10) | 48.7 (38.85-57.55) | 47.45 (34.12-62.88) | 0.632 |
| **Peripheral lymphocyte subset test** |  |  |  |  |
| B cells, % | 13 (5.32-22.08) | 8.93 (4-16.41) | 20.04 (10.98-26.01) | ＜0.001*** |
| T cells, % | 67.31 (59.67-75.94) | 66.9 (58.78-72.05) | 68.26 (61.05-79.8) | 0.059 |
| Th cells, mean (SD), % | 37.38 (11.5) | 34.12 (9.87) | 40.65 (12.14) | ＜0.001*** |
| Ts cells, % | 26 (19.38-33.9) | 28 (22.68-34.79) | 23.98 (18-32.97) | 0.059 |
| CD4/CD8 | 1.4 (0.95-2.08) | 1.23 (0.9-1.69) | 1.62 (1.04-2.68) | 0.003** |
| NK cells, % | 13.53 (8.58-23) | 21.99 (14.36-28) | 9 (6-12) | ＜0.001*** |
| B cells, cells/µL | 145.55 (73.53-279.75) | 141.3 (47.47-307) | 153.2 (93.77-254.48) | 0.436 |
| T cells, cells/µL | 859.5 (543.38-1336.65) | 1111.15 (747.02-1504.08) | 634.5 (411.75-991.88) | ＜0.001*** |
| Th cells, cells/µL | 443.55 (282.25-740.15) | 587.15 (375.35-809.78) | 354.7 (232.23-538.25) | ＜0.001*** |
| Ts cells, cells/µL | 326.95 (186.93-568.5) | 473.5 (291-633.28) | 244.65 (129.4-360.75) | ＜0.001*** |
| NK cells, cells/µL | 173.5 (87.97-318.85) | 319.1 (252.5-428.98) | 87.95 (56.62-132.9) | ＜0.001*** |
| Lymphocytes, ×10^9^/L | 1.2 (0.8-1.84) | 1.62 (1.1-2.02) | 0.86 (0.66-1.31) | ＜0.001*** |
| WBC, ×10^9^/L | 8.3 (6.38-11.06) | 9.2 (7.2-11.88) | 7.11 (5.19-9.22) | ＜0.001*** |
| Neutrophils, ×10^9^/L | 6 (4.4-8.08) | 6.64 (5.47-9.7) | 5.48 (3.84-7.9) | 0.005** |
| NLR | 4.72 (3.19-8.56) | 4.09 (3.01-6.46) | 6.75 (3.86-10.26) | 0.003** |
| **laboratory tests** |  |  |  |  |
| Ferritin, ng/mL | 222.75 (72.08-619.7) | 149.7 (63.43-446.85) | 399.55 (84.05-830.75) | 0.003** |
| LDH, IU/L | 259.5 (217.25-320) | 254 (202.25-315.75) | 268.5 (229.25-323) | 0.166 |
| CK, IU/L | 45 (30.75-76) | 44 (32.25-75.75) | 47 (30-74.75) | 0.925 |
| KL-6, IU/mL | 1375 (664-2417.75) | 1179.5 (597-2582.75) | 1383.5 (713.75-2238) | 0.723 |
| ALT, IU/L | 31 (16-58.25) | 23 (16-34.75) | 51 (29-80.75) | ＜0.001*** |
| AST, IU/L | 29 (20.75-46) | 25 (20-32.75) | 36.5 (23.25-64.5) | ＜0.001*** |
| CRP, mg/L | 2.5 (0.58-5.06) | 2.5 (0.71-9.34) | 2.5 (0.5-3.58) | 0.043* |
| ESR, mm/h | 16 (9-28) | 12.5 (7.25-22.75) | 19 (12-30.5) | 0.02* |
| **clinical manifestation, n (%)** |  |  |  |  |
| Fever | 23 (14.7) | 6 (7.7) | 17 (21.8) | 0.013 |
| Cough | 56 (35.9) | 28 (35.9) | 28 (35.9) | 1 |
| Heliotrope rash | 53 (34.0) | 15 (19.2) | 38 (48.7) | ＜0.001 |
| Gottron sign | 76 (48.7) | 28 (35.9) | 48 (61.5) | 0.001 |
| Mechanism’s hands | 37 (23.7) | 19 (24.4) | 18 (23.1) | 0.851 |
| Arthritis | 32 (20.5) | 17 (21.8) | 15 (19.2) | 0.692 |
| **Treatment** |  |  |  |  |
| antifibrotics, n (%) | 28 (17.9) | 13 (16.7) | 16 (20.5) | 0.537 |
| GC, n (%) | 156 (100) |  |  |  |
| maximum GC dose, mg/day | 40 (20-60) | 30 (11.25-47.5) | 60 (40-80) | ＜0.001*** |
| IVIG, n (%) | 13 (8.3) | 5 (6.4) | 8 (10.3) | 0.772 |
| Immunosuppressants or biologics, n (%) | 144 (92.3) | 68 (87.2) | 76 (97.4) | 0.016* |
| TAC | 26 (16.7) | 14 (17.9) | 12 (15.4) | 0.667 |
| HCQ | 3 (1.9) | 2 (2.6) | 1 (1.3) | 1 |
| CsA | 8 (5.1) | 5 (6.4) | 3 (3.8) | 0.717 |
| MMF | 4 (2.6) | 3 (3.8) | 1 (1.3) | 0.612 |
| CTX | 9 (5.8) | 2 (2.6) | 7 (9.0) | 0.17 |
| Agu | 2 (1.3) | 1 (1.3) | 1 (1.3) | 1 |
| MTX | 14 (9.0) | 12 (15.4) | 2 (2.6) | 0.005 |
| TOF | 87 (55.8) | 30 (38.5) | 57 (73.1) | ＜0.001*** |
| Baricitinib | 2 (1.3) | 0 (0) | 2 (2.6) | 0.477 |
| RTX | 21 (13.5) | 15 (19.2) | 6 (7.7) | 0.035 |
| tocilizumab | 4 (2.6) | 2 (2.6) | 2 (2.6) | 1 |

All but indicated PFTs results at endpoint show baseline characteristics. *p < 0.05; **p < 0.01; ***p < 0.001. Rapidly Progressive Interstitial Lung Disease (RP-ILD) is characterized by an accelerated deterioration in radiological interstitial changes, progressive dyspnea, and worsening hypoxemia, occurring within 1 month of onset of respiratory symptoms, irrespective of therapeutic interventions.

NSIP, nonspecific interstitial pneumonia, UIP, usual interstitial pneumonia, OP, organizing pneumonia; CK, creatine kinase; KL-6, Krebs von den Lungen-6; ALT, alanine transaminase; AST, aspartate transaminase; anti-ARS: anti-aminoacyl-tRNA synthetase; CRP, C-reactive protein; DM: dermatomyositis; ESR, erythrocyte sedimentation rate; antifibrotics, including nintedanib and pirfenidone; IVIG, intravenous immunoglobulin; immunosuppressants or biologics, including tacrolimus (TAC), cyclosporin A (CSA), and mycophenolate mofetil (MMF); Cyclophosphamide (CTX), azathioprine (AZA), methotrexate (MTX), tofacitinib (TOF), Baricitinib, Rituximab (RTX) and tocilizumab; LDH, lactate dehydrogenase; NLR, neutrophil-to-lymphocyte ratio; MSAs: Myositis-specific autoantibodies; anti-MDA5: anti–melanoma differentiation–associated protein 5; PFT: pulmonary function test; FVC% pred: percentage of predicted forced vital capacity; FEV1% pred: percentage of forced expiratory volume in the first second; DLCO% pred: percentage of the predicted diffusion capacity for carbon monoxide;

Supplementary Table S2 Patient characteristics: non-PF-ILD vs. PF-ILD group

| **characteristics** | **non-PF-ILD（n=91）** | **PF-ILD（n=65）** | **p value** |
| --- | --- | --- | --- |
|  |  |  |  |
| age, mean (SD), years | 52.4 (11.09) | 54.31 (11.07) | 0.29 |
| Male, n (%) | 28 (30.8) | 17 (26.2) | 0.53 |
| Course, month | 6 (3-14) | 4 (1-8) | 0.005** |
| Follow-up duration, month | 10 (10-12) | 6 (3-9) | ＜0.001*** |
| **disease subtype, n (%)** |  |  | 0.121 |
| ASS | 41 (45.1) | 19 (29.2) |  |
| Anti-MDA5^+^DM | 43 (47.3) | 41 (63.1) |  |
| others | 7 (7.7) | 5 (7.7) |  |
| **MSAs** |  |  |  |
| Anti-MDA5 | 43 (47.3) | 41 (63.1) | 0.051 |
| Anti-ARS | 41 (45.1) | 19 (29.2) | 0.045* |
| Anti-Ro52 | 29 (31.9) | 23 (35.4) | 0.646 |
| **HRCT pattern, n (%)** |  |  | 0.564 |
| NSIP | 71 (81.3) | 57 (87.7) |  |
| UIP | 15 (16.5) | 7 (10.8) |  |
| OP | 2 (2.2) | 1 (1.5) |  |
| **Fibrotic pattern, n (%)** | 34 (37.4) | 22 (33.8) | 0.652 |
| **PFT** |  |  |  |
| FVC % pred at baseline, mean (SD) | 67.74 (17.12) | 70.98 (18.47) | 0.267 |
| FEV1% pred at baseline, mean (SD) | 71.16 (16.99) | 71.04 (16.79) | 0.966 |
| DLCO% pred at baseline mean (SD) | 45.28 (18.17) | 44.79 (18.1) | 0.868 |
| FVC% pred at endpoint, mean (SD) | 76.89 (18.42) | 58.6 (16.32) | ＜0.001*** |
| FEV1% pred at endpoint, mean (SD) | 79.83 (18.98) | 61.01 (16.48) | ＜0.001*** |
| DLCO% pred at endpoint, % | 54 (42.95-68.45) | 38 (21-52) | ＜0.001*** |
| **Peripheral lymphocyte subset test** |  |  |  |
| B cells, % | 12.95 (5.12-21.26) | 14 (7-25.7) | 0.173 |
| T cells, % | 67.3 (59.96-74.37) | 68 (59.44-78) | 0.843 |
| Th cells, mean (SD), % | 37.4 (11.97) | 37.36 (10.91) | 0.985 |
| Ts cells, % | 26.69 (21-32) | 24.37 (18-36) | 0.657 |
| CD4/CD8 | 1.38 (0.96-1.99) | 1.42 (0.95-2.38) | 0.709 |
| NK cells, % | 15 (9.2-24.54) | 11 (7-17.3) | 0.014* |
| B cells, cells/µL | 166.5 (82.45-317.9) | 117 (57-205) | 0.045* |
| T cells, cells/µL | 1057.6 (750.05-1475.9) | 577 (398.2-961) | ＜0.001*** |
| Th cells, cells/µL | 600 (373-820.85) | 346 (238-457) | ＜0.001*** |
| Ts cells, cells/µL | 432.7 (253.4-598.2) | 250 (117-410.6) | ＜0.001*** |
| NK cells, cells/µL | 253.7 (153.7-370.4) | 100 (65-180.7) | ＜0.001*** |
| Lymphocytes, ×10^9^/L | 1.55 (1.01-2.02) | 0.86 (0.68-1.2) | ＜0.001*** |
| WBC, ×10^9^/L | 8.62 (6.72-11.87) | 7.37 (5.34-9.25) | 0.006** |
| Neutrophils, ×10^9^/L | 6.26 (4.9-9.16) | 5.66 (4.16-7.88) | 0.074 |
| NLR | 4.23 (2.96-7.27) | 5.45 (4.01-10.36) | 0.008** |
| **laboratory tests** |  |  |  |
| Ferritin, ng/mL | 148 (69.9-471.7) | 413.5 (104.7-776.8) | 0.012* |
| LDH, IU/L | 254 (208.5-315) | 271 (230-334) | 0.219 |
| CK, IU/L | 44 (31-77) | 47 (30-71) | 0.993 |
| KL-6, IU/mL | 1098 (616-2114.5) | 1419 (750-2585) | 0.289 |
| ALT, IU/L | 28 (15.5-43) | 41 (23-68) | 0.005** |
| AST, IU/L | 28 (20-38) | 36 (22-57) | 0.015* |
| CRP, mg/L | 2.5 (0.6-5.88) | 2.5 (0.55-4) | 0.586 |
| ESR, mm/h | 14 (8-23.5) | 19 (11-30) | 0.121 |
| **clinical manifestation, n (%)** |  |  |  |
| Fever | 12 (13.2) | 11 (16.9) | 0.516 |
| Cough | 29 (31.9) | 27 (41.5) | 0.214 |
| Heliotrope rash | 30 (33) | 23 (35.4) | 0.753 |
| Gottron sign | 40 (44) | 36 (55.4) | 0.159 |
| Mechanism’s hands | 22 (24.2) | 15 (23.1) | 0.874 |
| Arthritis | 22 (24.2) | 10 (15.4) | 0.18 |
| **Treatment** |  |  |  |
| antifibrotics | 17 (18.7) | 12 (18.5) | 0.972 |
| maximum GC dose, mg/day | 40 (16.25-70) | 50 (30-60) | 0.094 |
| IVIG, n (%) | 6 (6.6) | 7 (10.8) | 0.352 |
| Immunosuppressants or biologics, n (%) | 84 (92.3) | 60 (92.3) | 1 |
| TAC | 16 (17.6) | 10 (15.4) | 0.717 |
| HCQ | 2 (2.2) | 1 (1.5) | 1 |
| CsA | 3 (3.3) | 5 (7.7) | 0.39 |
| MMF | 3 (3.3) | 1 (1.5) | 0.864 |
| CTX | 6 (6.6) | 3 (4.6) | 0.862 |
| Agu | 0 (0) | 2 (3.1) | 0.336 |
| MTX | 9 (9.9) | 5 (7.7) | 0.636 |
| TOF | 48 (52.7) | 39 (60) | 0.369 |
| Baricitinib | 1 (1.1) | 1 (1.5) | 1 |
| RTX | 14 (15.4) | 7 (10.8) | 0.405 |
| tocilizumab | 2 (2.2) | 2 (3.1) | 1 |

Supplementary Table S3 univariate and multivariate regression analyses of risk factors for PF-ILD in total cohort

| **IIM (N=156)** | **Univariate Cox regression** | | **Multivariable Cox regression** | |
| --- | --- | --- | --- | --- |
|  | **HR (95%CI)** | **P value** | **HR (95%CI)** | **adjusted P value** |
| age, mean (SD), years | 1.026 (1.002-1.05) | 0.032* | 1.039 (1.012-1.066) | 0.004** |
| Male, n (%) | 0.876 (0.504-1.525) | 0.641 | 0.981 (0.542-1.777) | 0.95 |
| Course, month | 0.957 (0.919-0.997) | 0.036* | 0.986 (0.942-1.033) | 0.555 |
| **MSAs** |  |  |  |  |
| Anti-MDA5 | 1.634 (0.985-2.711) | 0.057 |  |  |
| Anti-ARS | 0.601 (0.352-1.028) | 0.063 |  |  |
| Anti-Ro52 | 1.151 (0.691-1.917) | 0.589 |  |  |
| **Peripheral lymphocyte subset test** |  |  |  |  |
| B cells, % | 1.022 (1-1.045) | 0.053 |  |  |
| T cells, % | 0.996 (0.977-1.016) | 0.724 |  |  |
| Th cells, mean (SD), % | 0.998 (0.978-1.019) | 0.868 |  |  |
| Ts cells, % | 0.997 (0.975-1.019) | 0.785 |  |  |
| CD4/CD8 | 1.163 (0.927-1.46) | 0.192 |  |  |
| NK cells, % | 0.983 (0.959-1.007) | 0.167 |  |  |
| B cells, cells/µL | 0.999 (0.998-1) | 0.115 | 1 (0.998-1.002) | 0.944 |
| T cells, cells/µL | 0.999 (0.998-0.999) | <0.001*** | 1.001 (0.997-1.004) | 0.758 |
| Th cells, cells/µL | 0.998 (0.997-0.999) | <0.001*** | 0.998 (0.994-1.001) | 0.196 |
| Ts cells, cells/µL | 0.998 (0.996-0.999) | <0.001*** | 0.999 (0.996-1.003) | 0.736 |
| NK cells, cells/µL | 0.995 (0.992-0.997) | <0.001*** | 0.995 (0.992-0.997) | ＜0.001*** |
| Lymphocytes, ×10^9^/L | 0.428 (0.28-0.655) | <0.001*** | 1.335 (0.484-3.684) | 0.577 |
| WBC, ×10^9^/L | 0.917 (0.855-0.983) | 0.015* | 0.956 (0.848-1.078) | 0.464 |
| Neutrophils, ×10^9^/L | 0.937 (0.866-1.013) | 0.102 |  |  |
| NLR | 1.065 (1.014-1.118) | 0.012* | 1.014 (0.911-1.129) | 0.795 |
| **laboratory tests** |  |  |  |  |
| Ferritin, ng/mL | 1 (1-1.001) | 0.083 |  |  |
| LDH, IU/L | 1 (0.999-1.002) | 0.689 |  |  |
| CK, IU/L | 1 (0.999-1.001) | 0.78 |  |  |
| KL-6, IU/mL | 1 (1-1) | 0.964 |  |  |
| ALT, IU/L | 1.001 (0.999-1.002) | 0.286 |  |  |
| AST, IU/L | 1 (0.999-1.001) | 0.804 |  |  |
| CRP, mg/L | 0.979 (0.946-1.012) | 0.215 |  |  |
| ESR, mm/h | 1.005 (0.994-1.016) | 0.362 |  |  |
| **Treatment** |  |  |  |  |
| antifibrotics | 0.909 (0.484-1.705) | 0.765 | 1.394 (0.62-3.133) | 0.421 |
| maximum GC dose, mg/day | 1.004 (0.998-1.01) | 0.173 | 0.996 (0.987-1.004) | 0.32 |
| IVIG, n (%) | 1.379 (0.628-3.031) | 0.424 | 1.089 (0.433-2.744) | 0.856 |
| Immunosuppressants or biologics, n (%) | 1.02 (0.409-2.546) | 0.966 | 0.512 (0.175-1.497) | 0.221 |

Supplementary Table S4 univariate and multivariate regression analyses of risk factors for PF-ILD in anti-MDA5^+^ DM patients

| ***anti-MDA5^+^ DM (N=84)*** | Univariate Cox regression | | Multivariable Cox regression | |
| --- | --- | --- | --- | --- |
|  | **HR (95%CI)** | P value | **HR (95%CI)** | adjusted P value |
| sex (vs female) | 0.795 (0.398-1.591) | 0.517 | 0.541 (0.238-1.23) | 0.143 |
| age ＞ 43.5 years | 2.869 (1.12-7.353) | 0.028* | 7.653 (2.005-29.204) | 0.003 |
| course (month) | 0.921 (0.856-0.991) | 0.029* | 0.983 (0.913-1.059) | 0.654 |
| **Peripheral lymphocyte subset test** |  |  |  |  |
| T cells ＜ 807.15 cells/μL | 3.873 (1.84-8.153) | <0.001*** |  |  |
| Th cells ＜ 533.2 cells/μL | 5.354 (1.905-15.052) | 0.001** | 4.703 (1.014-21.821) | 0.048 |
| Ts cells ＜ 186.85 cells/μL | 3.286 (1.747-6.181) | <0.001*** | 0.911 (0.289-2.873) | 0.874 |
| NK cells ＜ 148 cells/μL | 6.838 (2.668-17.525) | <0.001*** | 6.277 (1.572-25.067) | 0.009 |
| Lymphocytes ＜ 1.23×10^9^/L | 4.739 (1.849-12.149) | 0.001** | 0.484 (0.121-1.937) | 0.305 |
| NLR ＞ 3.921 | 2.863 (1.319-6.215) | 0.008** | 1.656 (0.657-4.173) | 0.285 |
| **Laboratory tests** |  |  |  |  |
| Ferritin ＞ 250.1 ng/mL | 2.677 (1.327-5.399) | 0.006** | 1.583 (0.644-3.895) | 0.317 |
| KL-6 ＞ 875.5 IU/mL | 2.045 (1.056-3.961) | 0.034* | 1.181 (0.55-2.54) | 0.669 |
| ALT ＞ 48 IU/L | 2.568 (1.367-4.827) | 0.003** | 1.875 (0.523-6.724) | 0.335 |
| AST ＞ 41.5 IU/L | 2.238 (1.211-4.139) | 0.01* | 0.3 (0.07-1.29) | 0.106 |
| ESR ＞ 17 mm/h | 2.188 (1.13-4.235) | 0.02* | 2.214 (0.63-7.776) | 0.215 |
| **Clinical manifestation** |  |  |  |  |
| Fever | 1.215 (0.577-2.561) | 0.609 |  |  |
| Cough | 1.044 (0.546-1.995) | 0.896 |  |  |
| Heliotrope | 0.804 (0.433-1.49) | 0.487 |  |  |
| Gottron | 1.489 (0.71-3.12) | 0.292 |  |  |
| Mechanism | 0.768 (0.339-1.74) | 0.527 |  |  |
| Arthritis | 0.776 (0.368-1.637) | 0.506 |  |  |
| **Treatment** |  |  |  |  |
| antifibrotics | 0.782 (0.345-1.771) | 0.555 | 1 (0.987-1.014) | 0.998 |
| GC | 1.003 (0.998-1.009) | 0.25 | 1.158 (0.35-3.838) | 0.81 |
| IVIG | 1.167 (0.455-2.998) | 0.748 | 1.087 (0.112-10.537) | 0.943 |
| Immunosuppressants | 73083530.935 (0-Inf) | 0.997 | 0.889 (0.22-3.596) | 0.869 |

Supplementary Table S5 univariate and multivariate regression analyses of risk factors for PF-ILD in ASS patients

| ***ASS (N=60)*** | Univariate Cox regression | | Multivariable Cox regression | |
| --- | --- | --- | --- | --- |
|  | **HR (95%CI)** | P value | **HR (95%CI)** | adjusted P value |
| sex (vs female) | 1.108 (0.391-3.137) | 0.847 | 0.862 (0.218-3.41) | 0.833 |
| age | 1.016 (0.974-1.06) | 0.46 | 0.983 (0.926-1.043) | 0.564 |
| course (month) | 0.998 (0.943-1.056) | 0.938 | 0.898 (0.787-1.025) | 0.11 |
| **Peripheral lymphocyte subset test** |  |  |  |  |
| B cells ＜ 209.15 cells/µL | 5.906 (1.352-25.811) | 0.018* | 5.069 (0.543-47.304) | 0.154 |
| T cells ＜ 725.35 cells/µL | 2.541 (1.015-6.36) | 0.046* | 0.263 (0.05-1.381) | 0.114 |
| Th cells ＜ 430.85 cells/µL | 2.512 (0.983-6.418) | 0.054 |  |  |
| Ts cells ＜ 516.5 cells/µL | 2.089 (0.687-6.357) | 0.194 |  |  |
| NK cells ＜ 303.3 cells/µL | 5.816 (1.687-20.053) | 0.005** | 19.962 (3.108-128.223) | 0.002 |
| Lymphocytes ＜ 1.545×10^9^/L | 3.381 (1.213-9.426) | 0.02* | 9.684 (1.063-88.186) | 0.044 |
| NLR ＞ 9.181 | 1.853 (0.733-4.682) | 0.192 |  |  |
| **Laboratory tests** |  |  |  |  |
| Ferritin ＞ 259.45 ng/mL | 4.787 (1.778-12.89) | 0.002** | 6 (1.116-32.256) | 0.037 |
| LDH ＞ 296.5 IU/L | 2.704 (1.095-6.674) | 0.031* | 0.503 (0.081-3.137) | 0.462 |
| ALT ＞ 30.5 IU/L | 1.73 (0.694-4.313) | 0.24 |  |  |
| AST ＞ 35.5 IU/L | 3.225 (1.262-8.24) | 0.014* | 2.571 (0.719-9.187) | 0.146 |
| **Clinical manifestation** |  |  |  |  |
| Fever | 0.415 (0.054-3.162) | 0.396 |  |  |
| Cough | 0.982 (0.384-2.506) | 0.969 |  |  |
| Heliotrope | 0.925 (0.209-4.087) | 0.918 |  |  |
| Gottron | 0.692 (0.201-2.384) | 0.559 |  |  |
| Mechanism | 1.436 (0.555-3.714) | 0.456 |  |  |
| Arthritis | 0.245 (0.032-1.849) | 0.172 |  |  |
| **Treatment** |  |  |  |  |
| antifibrotics | 1.27 (0.416-3.877) | 0.675 | 1.016 (0.986-1.047) | 0.309 |
| GC | 0.999 (0.981-1.017) | 0.922 | 2.351 (0.356-15.504) | 0.375 |
| IVIG | 2.394 (0.548-10.45) | 0.246 | 0.041 (0.005-0.321) | 0.002 |
| Immunosuppressants | 0.358 (0.117-1.091) | 0.071 | 2.274 (0.223-23.186) | 0.488 |
